# Pioglitazone Synthetic Analogue Ameliorates Streptozotocin-Induced Diabetes Mellitus through Modulation of ACE 2/Angiotensin 1–7 via PI3K/AKT/mTOR Signaling Pathway

**DOI:** 10.3390/ph15030341

**Published:** 2022-03-10

**Authors:** Yasmin M. Ahmed, Mohamed A. Abdelgawad, Khaled Shalaby, Mohammed M. Ghoneim, Asmaa M. AboulMagd, Nada S. Abdelwahab, Hossam M. Hassan, Asmaa M. Othman

**Affiliations:** 1 Department of Pharmacology and Toxicology, Faculty of Pharmacy, Nahda University, Beni-Suef 62514, Egypt; yasmain.mostafa@nub.edu.eg; 2 Department of Pharmaceutical Chemistry, College of Pharmacy, Jouf University, Sakaka 72341, Al Jouf, Saudi Arabia; 3 Department of Pharmaceutics, College of Pharmacy, Jouf University, Sakaka 72341, Al Jouf, Saudi Arabia; khshalabi@ju.edu.sa; 4 Department of Pharmacy Practice, College of Pharmacy, AlMaarefa University, Ad Diriyah 13713, Saudi Arabia; mghoneim@mcst.edu.sa; 5 Department of Pharmaceutical Chemistry, Faculty of Pharmacy, Nahda University, Beni-Suef 62514, Egypt; nada.abdelwahab@nub.edu.eg; 6 Department of Pharmaceutical Analytical Chemistry, Faculty of Pharmacy, Beni-Suef University, Beni-Suef 62521, Egypt; 7 Department of Pharmacognosy, Faculty of Pharmacy, Beni-Suef University, Beni-Suef 62521, Egypt; hossam.mokhtar@nub.edu.eg; 8 Department of Internal Medicine, Faculty of Medicine, Beni-Suef University, Beni-Suef 62514, Egypt; assmaa_med@yahoo.com

**Keywords:** angiotensin-converting enzyme 2 (ACE 2), angiotensin 1–7, liver, insulin sensitivity, type 2 diabetes mellitus, phosphoinositide 3-kinases (PI3k), serein/threonine kinase (AKT)

## Abstract

The renin angiotensin aldosterone system has a localized key regulatory action, especially in liver and body circulation. Furthermore, it accomplishes a significant role in the downregulation of the PI3K/AKT/mTOR signaling pathway that is involved in type II diabetes mellitus pathogenesis. The current study aimed to evaluate the effect of a synthetic pioglitazone analogue (benzenesulfonamide derivative) compared to the standard pioglitazone hypoglycemic drug on enhancing liver insulin sensitivity via ACE 2/Ang (1–7)/PI3K/AKT/mTOR in experimental STZ-induced diabetes. After the model was established, rats were distributed into the normal control group, diabetic group, pioglitazone group (20 mg/kg), and a benzenesulfonamide derivative group (20 mg/kg), with the last 2 groups receiving oral treatment for 14 consecutive days. Our results suggested enhancing liver insulin sensitivity against the ACE2/Ang (1–7)/PI3K/AKT/mTOR pathway. Moreover, the synthetic compound produced a reduction in blood glucose levels, restored hyperinsulinemia back to normal, and enhanced liver glycogen deposition. In addition, it up regulated the ACE2/Ang (1–7)/PI3K/AKT/mTOR signaling pathway via increasing insulin receptor substrate 1 and 2 sensitivity to insulin, while it increased glucose transporter 2 expression in the rat pancreas. The study findings imply that the hypoglycemic effect of the benzenesulfonamide derivative is due to enhancing liver sensitivity to regulate blood glucose level via the ACE2/Ang (1–7)/PI3K/AKT/mTOR pathway.

## 1. Introduction

Hyperglycemia is a term used to describe a spectrum of metabolic disorders characterized by elevated blood glucose levels caused by abnormalities in insulin secretion, insulin sensitivity, or both [1]. Type 1 diabetes mellitus, in which the pancreas beta cells fail to produce insulin, referred to as “insulin-dependent diabetes mellitus”, and type 2 diabetes mellitus, in which the insulin receptor sites do not respond to insulin, referred to as “non-insulin-dependent diabetes mellitus”, are the two most common diseases that cause hyperglycemia [2]. Diabetes is expected to become an epidemic as the global population grows from 150 million to 300 million by 2025, especially type 2, which is closely linked to obesity and a sedentary lifestyle and will account for the great majority of cases [3]. Additionally, insulin resistance is a diagnostic sign for obesity-related diabetes mellitus, and it is regarded as the main risk factor for physiological phenomena in which organs such as the liver, skeletal muscle, and adipose tissue are less receptive to insulin [4,5].

In the same context, insulin resistance is assumed to be induced primarily by irregular hepatic insulin action, in which higher insulin levels are required to control blood glucose levels [6]. Additionally, liver and white adipose tissues are the most abundant source of angiotensinogen and leptin in which both are insulin-sensitive regulators [7,8]. The role of the localized renin-angiotensin system (RAS) in the evolution of many pathological disorders has been hypothesized based on accumulating evidence [9]. The oligopeptide hormone angiotensin II, one of the main regulatory RAS components, was implicated in the etiology of diabetes and its co-morbidities [10]. When angiotensin-converting enzyme (ACE) is replaced with (ACE 2), a main product for angiotensin (1–7) instead of Ang II, a biological antagonist to the conventional RAS exists [11]. In contrast to the traditional RAS system, stimulation of the Ang II-Ang (1–7) axis has been shown to have powerful antioxidant and anti-inflammatory effects [12]. On the other hand, the RAS and leptin signaling pathway has been investigated to have linked cross talk through regulating glucose metabolism [13].

Furthermore, both IRS 1 and IRS 2 are widely expressed in hepatocytes; IRS 1 may be more responsible for glucose management, while IRS 2 may govern lipid handling [14]. In the presence of insulin, the downstream phosphoinositide 3-kinase (PI3K) signaling cascade is activated, leading to the activation of Akt and other downstream transmitters, such as IB kinase, ERK, JNK, PiK3, and mTOR, which negatively phosphorylate IRS proteins on their specific sites and inhibit their activity [8,9,10,11,12,13,14,15]. The activation of the PI3K/AKT/mTOR intracellular signaling pathway resulted in the translocation of insulin-regulated glucose transporter-4 (GLUT 4) into the plasma protein, facilitating glucose absorption through insulin response sites. Glycogen synthase kinase 3 is inhibited by IRS activation, which promotes glycogen production in both the liver and adipose tissue [16]. In quintessence, chronically high insulin levels in the blood cause inhibition of insulin signaling in IR-expressing tissues, resulting in insulin resistance in the insulin-sensitive tissues, such as the pancreas, liver, muscle, and fat, as well as in insulin-insensitive tissues, such as the brain, macrophages, and vascular endothelial cells [17,18].

Current type 2 DM treatment protocols are based on a non-pharmacological portion through balanced diet and weight management as well pharmacological treatment by increasing insulin sensitivity on its target site using metformin, glitazones, sulfonylurea, α-glucosidase inhibitors, and dipeptidyl peptidase-4 inhibitors [19,20]. Consequently, novel therapeutic treatments for type 2 diabetes are urgently needed.

We hypothesized that the new synthetic oral hypoglycemic would increase liver insulin sensitivity due to the activation of the ACE 2/Ang (1–7)/PI3K/AKT/mTOR axis on experimentally high-fat diet-induced diabetes in albino Wistar rats. To achieve this goal, serum ELISA insulin levels were measured as well as ACE 2, Ang (1–7), IRS-1, IRS-2, leptin, GLP-1, IL-1β, and glycogen using ELISA liver tissue homogenate. Furthermore, Western blot analysis was used to estimate the p-PI3k/p-AKT/p-mTOR signaling pathway. In order to confirm our theory, a histological H&E examination of the liver and an immunohistochemistry investigation of pancreas tissue GLUT 2 were performed.

## 2. Results

### 2.1. Biochemical Estimation Regarding ELISA

#### 2.1.1. Plasma Fasting Insulin Levels concerning Type 2 Diabetes Mellitus Induction

**Figure 1:**
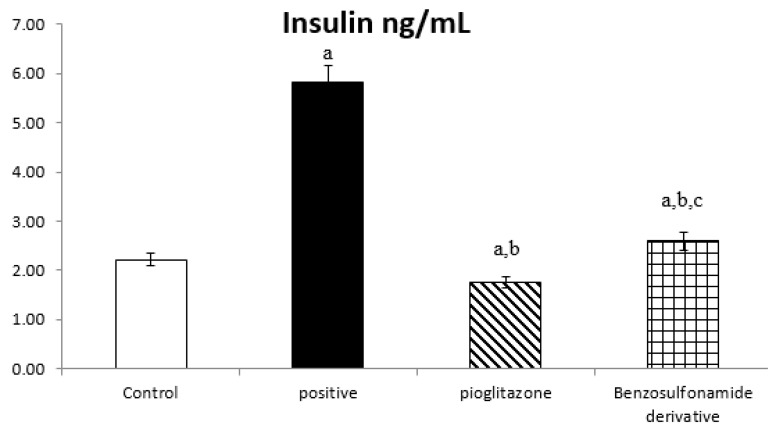
Bar chart presentation of fasting serum insulin concerning the effect of 14 days of oral administration of the synthetic compound against the reference anti-hyperglycemic pioglitazone on STZ-induced type 2 DM. DM: diabetes mellitus. Each bar represents the mean of 6–8 values ± the standard error of the mean (S.E.M). a: Significantly different from the normal control group, b: Significantly different from the positive control group, c: Significantly different from the pioglitazone group at *p* < 0.05.

The serum fasted insulin results for the normal control rats was 2.22 ± 0.11 ng/mL. Rats subjected to HFD and STZ administration, single dose of 45 mg/kg (positive control group), showed a significant increase in insulin levels to 5.83 ± 0.31 ng/mL. compared to the normal control group. The treatment of diabetic rats with the standard pioglitazone hypoglycemic drug significantly decreased insulin levels, as they were 1.75 ± 0.11 ng/mL compared to the positive control group. Additionally, the treatment of diabetic rats with the benzenesulfonamide derivative (new synthetic compound) showed a significant decrease in serum fasted insulin levels to 2.59 ± 0.17 ng/mL compared to the positive control group. Moreover, it restored insulin levels to normal when compared to the control rats and the standard treatment group (Figure 1).

#### 2.1.2. Activation of ACE 2 and Ang (1–7) Results in Increased Glycogen Synthesis in Hepatocyte Tissues

**Figure 2:**
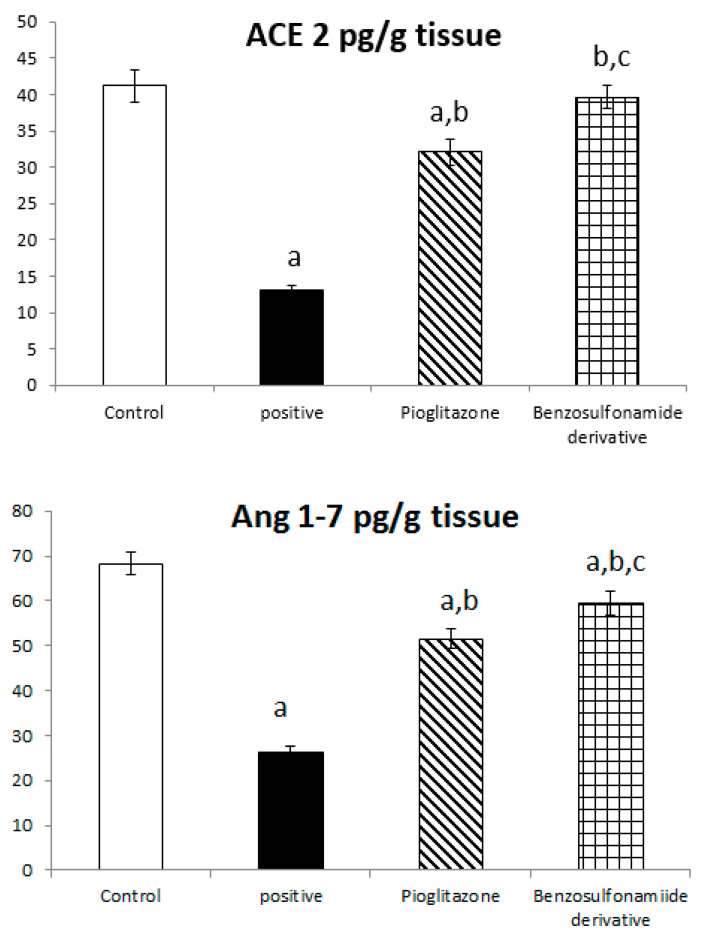

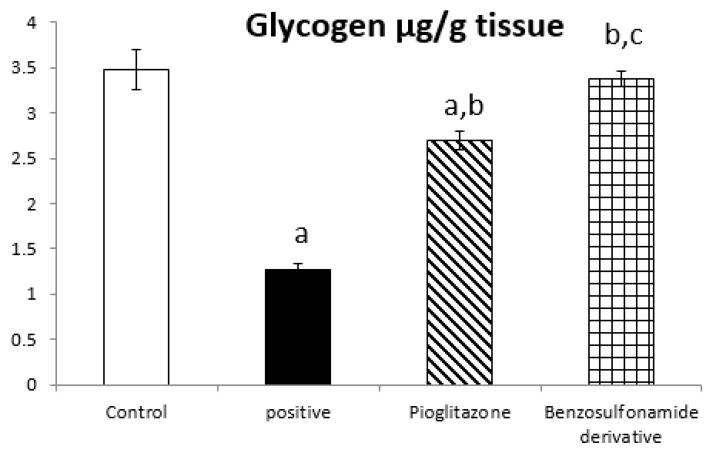
Bar chart presentation of liver tissue ACE 2, Ang (1–7), and glycogen concerning the effect of 14 days of oral administration of the synthetic compound against the reference anti-hyperglycemic pioglitazone on STZ-induced type 2 DM. DM: diabetes mellitus. Each bar represents the mean of 6–8 values ± the standard error of the mean (S.E.M). a: Significantly different from the normal control group, b: Significantly different from the positive control group, c: Significantly different from the pioglitazone group at *p* < 0.05.

Both ACE 2 and Ang (1–7) are expressed in the hepatocyte tissues of normal rats as 41.17 ± 2.26 Pg/g and 68.33 ± 2.59 Pg/g, respectively. While rats subjected to HFD and STZ administration, single dose of 45 mg/kg (positive control group), showed a significant decrease in ACE 2 and Ang (1–7) to 13.10 ± 0.72 Pg/g and 26.36 ± 1.22 Pg/g, respectively, when compared to the normal control rats, treatment of diabetic rats with standard pioglitazone hypoglycemic drug significantly increased ACE 2 and Ang (1–7) activities to 32.10 ± 1.74 Pg/g and 51.60 ± 2.04 Pg/g, respectively, in comparison to the positive control group. Besides, the treatment of diabetic rats with the benzenesulfonamide derivative showed a significant increase in ACE 2 and Ang (1–7) levels, which reached 39.66 ± 1.54 Pg/g and 59.52 ± 2.57 Pg/g, respectively, compared to the positive control group and restored ACE 2 back to normal when compared to the normal control group. Moreover, it significantly increased ACE 2 and Ang (1–7) activity compared to the standard group (Figure 2).

On the other hand, the glycogen content in liver tissue was affected by ACE 2 and Ang (1–7) activities changes, as in normal rats was 3.48 ± 0.22 µg/g. The positive control group rats showed a significant decrease in glycogen content to 1.27 ± 0.17 µg/g compared to the normal control rats. The treatment of diabetic rats with the standard pioglitazone hypoglycemic drug significantly increased the glycogen content to 2.70 ± 0.10 µg/g compared to the positive control group. Additionally, the treatment of diabetic rats with the benzenesulfonamide derivative showed a significantly restored glycogen content back to a normal value of 3.37 ± 0.19 µg/g compared to the positive control group. Moreover, it significantly increased the glycogen content compared to the standard group (Figure 2).

#### 2.1.3. Activation of the ACE 2/Ang (1–7) Alters the Glucose Metabolism of IRS 1/IRS 2 in Hepatocyte Tissues

**Figure 3:**
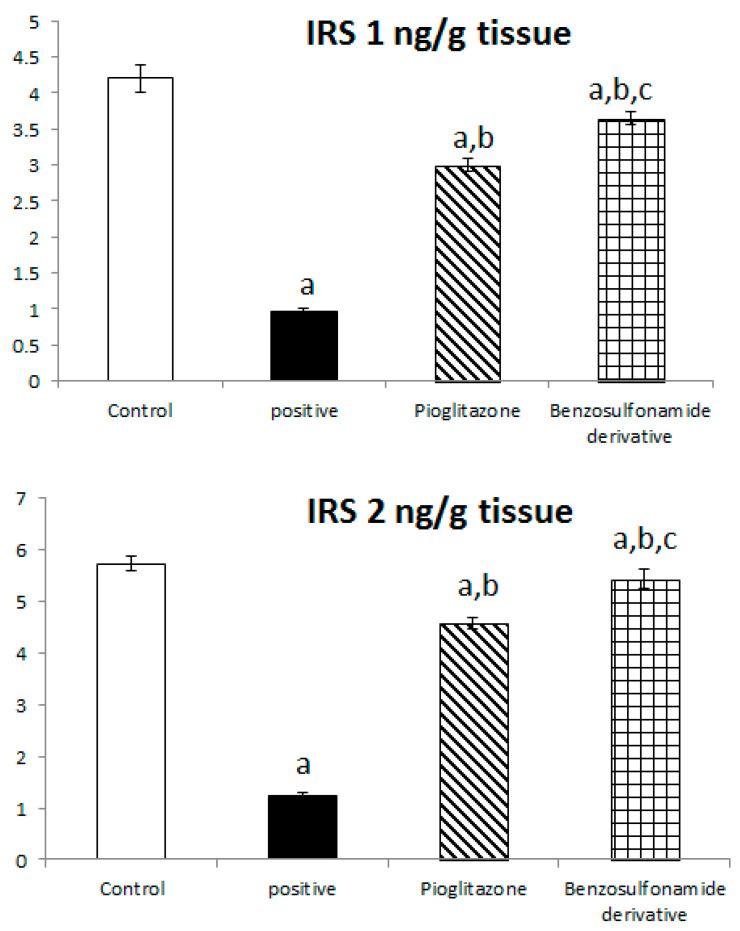
Bar chart presentation of liver tissue IRS-1 and IRS-2 concerning the effect of 14 days of oral administration of the synthetic compound against reference anti-hyperglycemic pioglitazone on STZ-induced type 2 DM. DM: diabetes mellitus. Each bar represents the mean of 6–8 values ± the standard error of the mean (S.E.M). a: Significantly different from the normal control group, b: Significantly different from the positive control group, c: Significantly different from the pioglitazone group at *p* < 0.05.

To further explore the role of ACE 2/Ang (1–7) to increase liver insulin sensitivity and glucose uptake, we investigated the liver tissue levels of IRS 1 protein which are responsible for glucose metabolism, and we found that in normal rats the IRS-1 and IRS 2 activities were 4.20 ± 0.19 ng/g and 5.72 ± 0.15 ng/g, respectively. Diabetic rats showed a significant decrease in IRS-1 and IRS-2 activities, which reached 0.96 ± 0.06 ng/g and 1.24 ± 0.07 ng/g, respectively, when compared to the normal control rats. The treatment of diabetic rats with the standard pioglitazone hypoglycemic drug significantly increased IRS-1 and IRS-2 activities to 3.00 ± 0.08 ng/g and 4.58 ± 0.12 ng/g, respectively, in comparison to the positive control group. The treatment of diabetic rats with the benzenesulfonamide derivative showed a significant increase in IRS 1 and IRS 2 levels, which reached 3.65 ± 0.09 ng/mL and 5.44 ± 0.19 ng/g, respectively, compared to the positive control group (Figure 3).

#### 2.1.4. Effect of ACE 2/Ang (1–7) on Leukocyte Endogenous Mediator IL-1β in Hepatocyte Tissues

**Figure 4:**
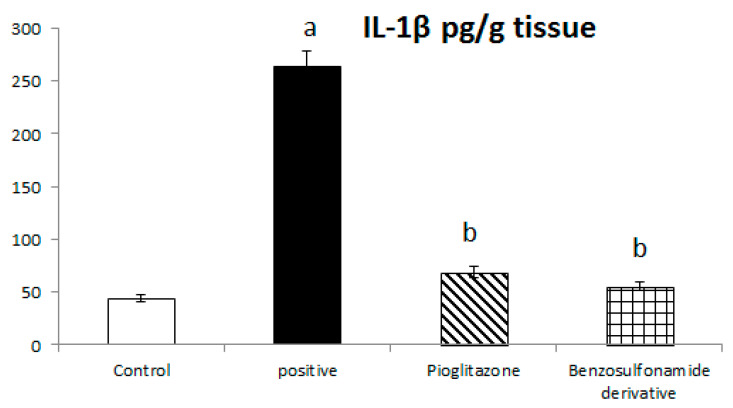
Bar chart presentation of liver tissue IL-1β concerning the effect of 14 days of oral administration of the synthetic compound against the reference anti-hyperglycemic pioglitazone on STZ-induced type 2 DM. DM: diabetes mellitus. Each bar represents the mean of 6–8 values ± the standard error of the mean (S.E.M). a: Significantly different from the normal control group, b: Significantly different from the positive control group.

The inhibition of ACE 2/Ang (1–7) by STZ was confirmed in Figure 1 to significantly stimulate the increase of IL-1β, as the normal control rat levels of IL-1β were 44.06 ± 3.58 Pg/g. The positive control rats showed a significant increase in IL-1β levels, which reached 263.94 ± 14.61 Pg/g, compared to the normal control rats. The treatment of diabetic rats with the standard pioglitazone hypoglycemic drug significantly decreased IL-1β to 68.80 ± 5.46 Pg/g in comparison to the positive control group. The treatment of diabetic rats with the benzenesulfonamide derivative showed significantly decreased IL-1β levels to 56.12 ± 4.03 Pg/g compared to positive control group (Figure 4).

#### 2.1.5. Hepatocyte Response to GLP-1 and Leptin

**Figure 5:**
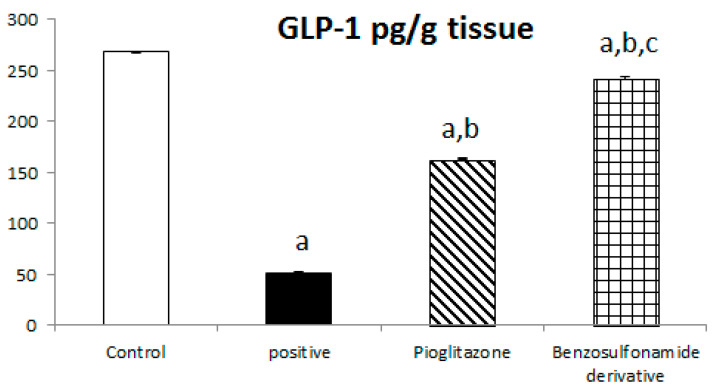

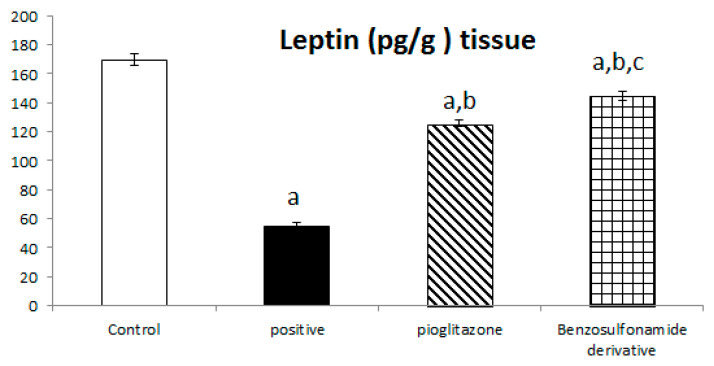
Bar chart presentation of liver tissue GLP-1 and leptin concerning the effect of 14 days of oral administration of the synthetic compound against the reference anti-hyperglycemic pioglitazone on STZ-induced type 2 DM. DM: diabetes mellitus. Each bar represents the mean of 6–8 values ± the standard error of the mean (S.E.M). a: Significantly different from the normal control group, b: Significantly different from the positive control group, c: Significantly different from the pioglitazone group at *p* < 0.05.

The incretin hormone glucagon-like peptide 1 (GLP1) potentiates insulin release, suppresses glucagon secretion, and enhances glycogen synthesis and deposition in the liver. Thus, normal control rat levels of GLP-1 and leptin were 267.56 ± 6.25 Pg/g and 168.63 ± 4.11 Pg/g, respectively. The positive control rats showed significantly decreased GLP-1 and leptin levels, which reached 51.51 ± 5.51 Pg/g and 54.97 ± 2.85 Pg/g, respectively, compared to the normal control rats. The treatment of diabetic rats with the standard pioglitazone hypoglycemic drug significantly increased GLP-1 and leptin levels to 162.75 ± 10.89 Pg/g and 125.80 ± 2.45 Pg/g, respectively, in comparison to the positive control group. The treatment of diabetic rats with the benzenesulfonamide derivative showed significantly increased GLP-1 and leptin levels of 242.30 ± 14.77 Pg/g and 144.72 ± 3.31 Pg/g, respectively, in comparison to the positive control group (Figure 5).

#### 2.1.6. ACE 2/Ang (1–7) Regulate the Expression of the PI3K/AKT/mTOR Signaling Pathway in the Liver

**Figure 6:**
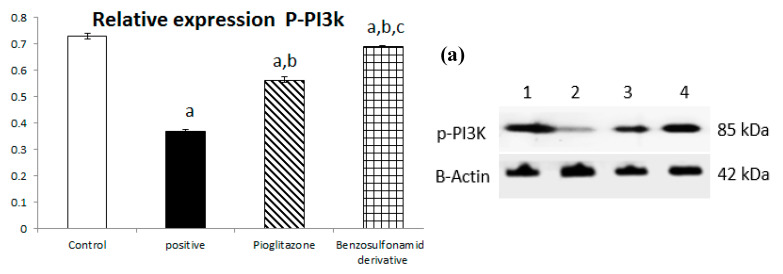

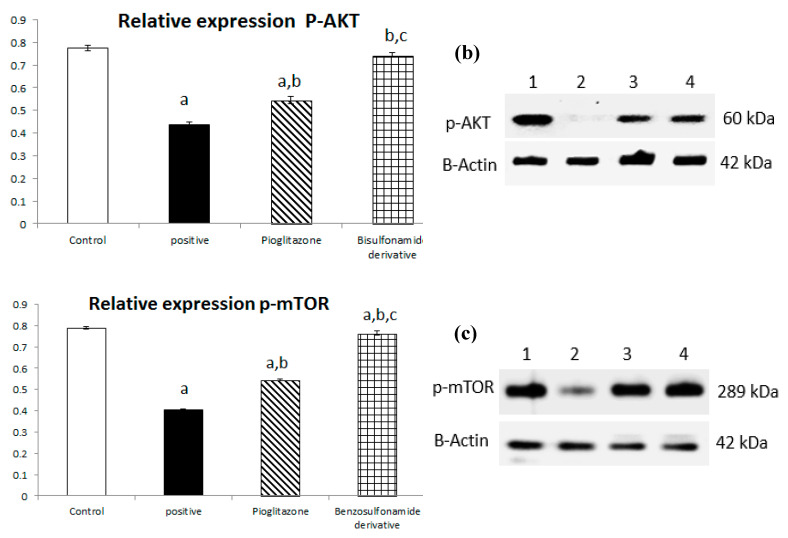
Bar chart presentation of the liver protein relative expression of p-PI3K/p-AKT/p-mTOR, figure subdivided into (**a**) represents p-PI3K, (**b**) represents p-AKT and (**c**) represents p-mTOR liver protein relative expression concerning the effect of 14 days of oral administration of the syn-thetic compound against the reference anti-hyperglycemic pioglitazone on STZ-induced type 2 DM. DM: diabetes mellitus. Each bar represents the mean of 6–8 values ± the standard error of the mean (S.E.M). a: Significantly different from the normal control group, b: Significantly different from the positive control group, c: Significantly different from the pioglitazone group at *p* < 0.05.

The downregulation of ACE 2/Ang (1–7) with STZ induction shown in Figure 1 has a directly proportional effect on the liver PI3K/AKT/mTOR signaling pathway. First, STZ induction significantly decreased p-PI3k relative protein expression by 50% when compared to the normal control group, and pioglitazone treatment upregulated relative protein expression by 152.76% compared to the STZ group, while the synthetic compound significantly up regulated p-PI3K relative protein expression by 187.5% compared to the STZ group (Figure 6a).

Additionally, the STZ group significantly downregulated p-AKT relative protein expression by 56.36% when compared to the normal group, and treatment with pioglitazone and the new synthetic compound both significantly upregulated p-AKT relative protein expression by 124.58% and 170.03%, respectively (Figure 6b).

In turn, p-mTOR relative protein expression was significantly downregulated through STZ induction by 51.46%, and treatment with pioglitazone and the new synthetic compound both significantly upregulated p-mTOR relative protein expression by 134.83% and 154.31%, respectively (Figure 6c).

#### 2.1.7. Histopathology

##### Photomicrographs of Rat Liver Sections Stained with H&E Showing the Effect of 14 Days of Oral Administration of Pioglitazone and the Synthetic Compound against STZ-Induced Type 2 DM

**Figure 7:**
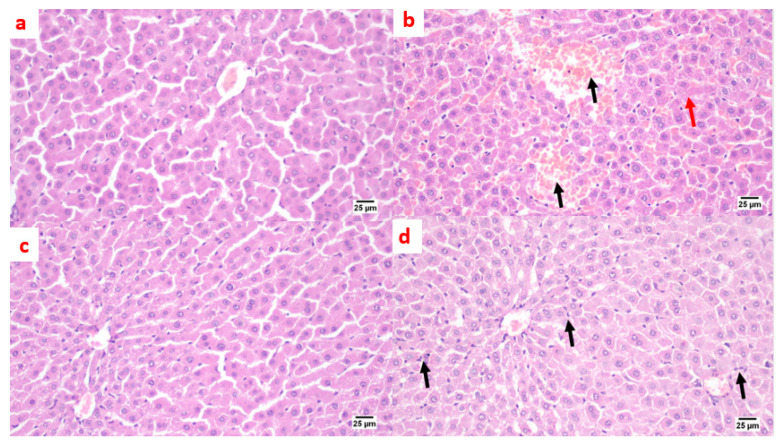
Photomicrographs of rat liver sections stained with H&E showing the effect of 14 days of oral administration of pioglitazone and the synthetic compound against STZ-induced type 2 DM. (**a**) Normal control section (25 µm). It appears normal histologically. The hepatocytes were arranged in parallel cords radiating from the central vein toward the portal area that contained branches of the portal vein, hepatic artery, and bile duct. On the contrary, (**b**) the positive control group section (25 µm) shows mononuclear inflammatory cell infiltration at the portal area with a hemorrhage (black arrow), a hyperplastic bile duct, sinusoidal dilatation, and a few necrotic hepatocytes (red arrow). (**c**) The pioglitazone-treated section (25 µm) shows the hepatic parenchyma was normal, without any detectable histopathological alterations. (**d**) The synthetic compound section (25 µm) shows a normal hepatic parenchyma with mild hyperplasia in the Kupffer cells, with mononuclear inflammatory cells infiltration at the portal area (black arrows).

A microscopic examination of liver tissue showed a normal histology. The hepatocytes were arranged in parallel cords radiating from the central vein toward the portal area that contained branches of the portal vein, hepatic artery, and bile duct (Figure 7a). On the contrary, in the positive control group, liver tissue exhibited some histopathological alterations; the portal areas were mildly expanded by mild fibroplasia and mononuclear inflammatory cell infiltration. The bile ducts within the portal areas were hyperplastic, and the portal blood vessels were severely congested. Focal areas of parenchymal loss were observed with hemorrhages and marked sinusoidal dilatation. Moreover, some hepatocytes suffered sporadic cell necrosis (Figure 7b). Regarding the treatment with pioglitazone, the hepatic parenchyma was normal, without any detectable histopathological alterations (Figure 7c). The livers of the benzenesulfonamide-derivative-treated synthetic group showed apparently normal hepatic parenchyma in almost all examined sections. Few sections showed mild hyperplasia in the Kupffer cells, with mononuclear inflammatory cells infiltration at the portal area. (Figure 7d).

##### Photomicrographs of Rat Pancreatic Sections Stained with H&E Showing the Effect of 14 Days of Oral Administration of Pioglitazone and the Synthetic Compound against STZ-Induced Type 2 DM

**Figure 8:**
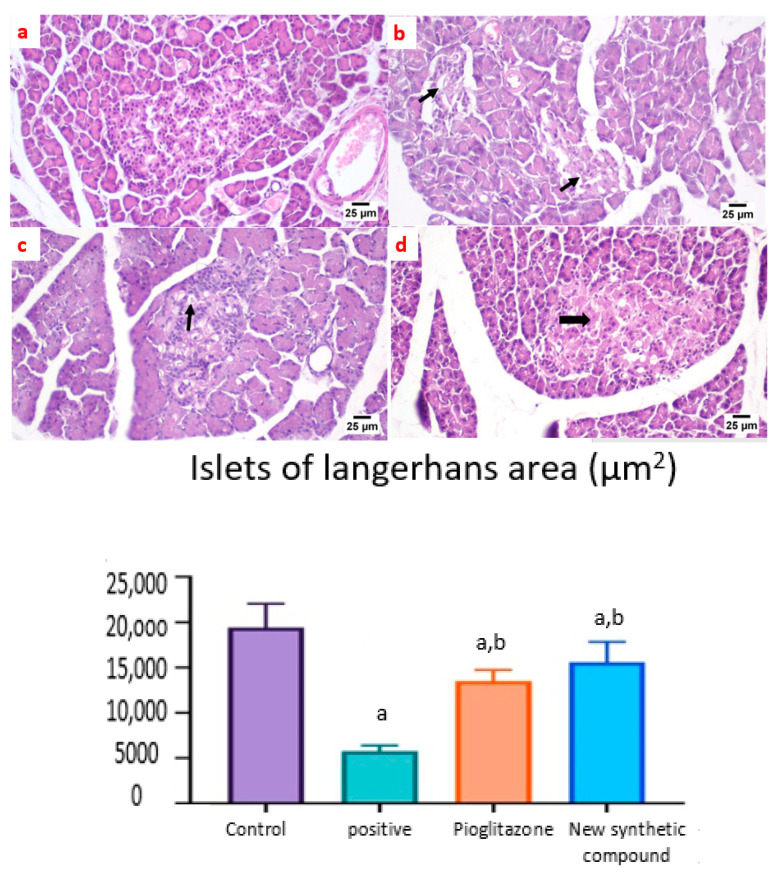
Photomicrographs of rat pancreatic sections stained with H&E showing the effect of 14 days of oral administration of pioglitazone and the synthetic compound against STZ-induced type 2 DM. (**a**) Normal control section (25 µm) showing the normal structures of both exocrine units and endocrine components of the pancreas. The islets of Langerhans appeared to be of normal size and contained β-cells; (**b**) positive control group section (25 µm) showing atrophied ill-distinct islets of Langerhans that contained few vacuolated and necrotic β-cells. Some of the examined sections exhibited inflammatory reactions in the peri-pancreatic tissue (black arrows); (**c**) pioglitazone treatment section (25 µm) showing variable sized islets that showed few vacuolated cells. Some sections exhibited apparently normal endocrine components; (**d**) synthetic compound section (25 µm) revealed normal structures of both the exocrine units and endocrine components of the pancreas. The islets of Langerhans appeared to be of normal size and contained β-cells (black arrow). The Langerhans islets area was decreased significantly in control positive group when compared to the other groups. Both pioglitazone and benzosulfonamide derivative groups showed significant increase in the islets in comparison with positive control group. Data were presented as mean ±SE. a: Significantly different from the normal control group, b: Significantly different from the positive control group at *p* < 0.05.

A microscopic examination of the pancreas from the normal control group and synthetic compound treatment group revealed a normal structure of both exocrine units and endocrine components of the pancreas. Additionally, the islets of Langerhans appeared to be of normal size and contained β-cells (Figure 8a,d). The positive control group showed atrophied ill-distinct islets of Langerhans that contained few vacuolated and necrotic β-cells. Some of the examined sections exhibited inflammatory reactions in the peri-pancreatic tissue (Figure 8b). The pancreas after standard pioglitazone hypoglycemic drug showed variable sized islets that showed few vacuolated cells. Some sections exhibited apparently normal endocrine components (Figure 8c).

**Figure 9:**
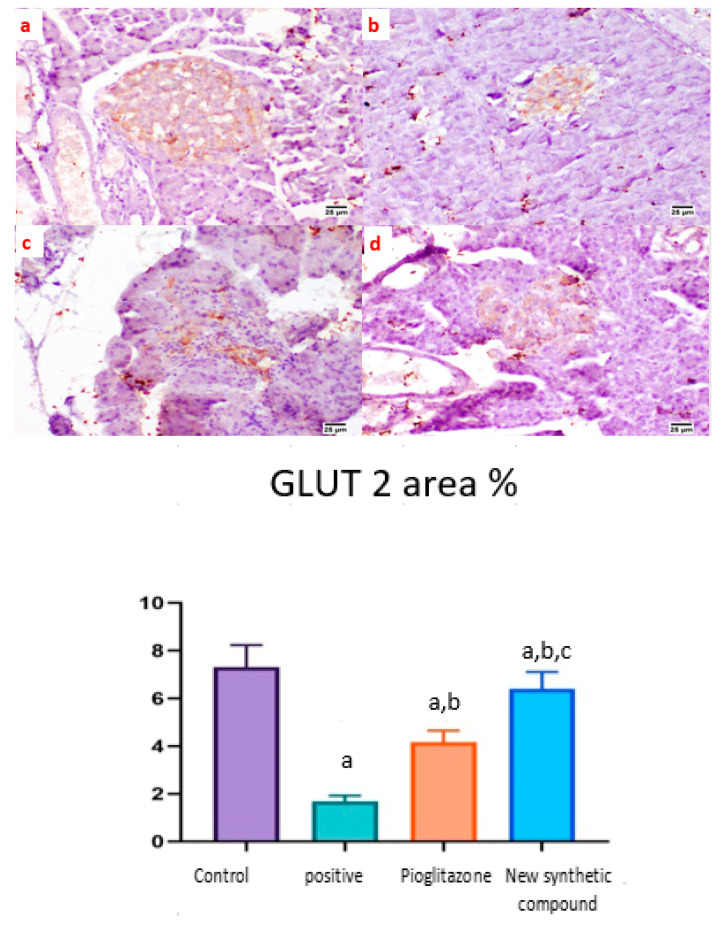
Photomicrographs of rat pancreatic immunostained section for glucose transporter 2 (GLUT 2) showing the effect of 14 days of oral administration of pioglitazone and the synthetic compound against STZ-induced type 2 DM. (**a**) Normal control section (25 µm) showing a high expression of GLUT-2 in pancreatic islets of Langerhans; (**b**) positive control group section (25 µm) showing limited expression of GLUT-2 in the islets of Langerhans; (**c**) pioglitazone treatment section (25 µm) showing increased expression of GLUT-2 in the islets of Langerhans; (**d**) synthetic compound section (25 µm) showing positive expression of GLUT-2 in the islets of Langerhans. The statistical analysis showed a significant increase in the normal, pioglitazone, and benzosulfonamide derivative in comparison with the positive control group. On the contrary, GLUT 2 % statistical analysis showed a significant increase in normal control group, pioglitazone standard group and benzosulfonamide derivative in comparison to positive control group. While, benzosulfonamide derivative groups showed significant increase in GLUT 2% in compared to positive control and pioglitazone groups (Figure 9). Immune expression of GLUT 2 as area %. Data are presented as means ± SE. Significant difference was considered at *p* > 0.05. a: Significantly different from the normal control group, b: Significantly different from the positive control group, c: Significantly different from the pioglitazone group at *p* < 0.05.

The expression of GLUT 2 was detected in the islets of Langerhans of different groups. Both the normal and synthetic compound treatment groups showed a strong positive staining in the examined islets (Figure 9a,d). Meanwhile, weak expression was noticed in the positive control group (Figure 9b). A higher expression was noticed in the standard pioglitazone-treated group among the different examined sections (Figure 9c).

## 3. Discussion

Our work revealed the effect of the thiazolidinedione’s pioglitazone and benzosulfonamide derivative that were investigated against STZ- and HFD-induced type 2 diabetes mellitus in rats.

The primary cause of type 2 diabetes is thought to be hepatic insulin resistance [21]. The renin–angiotensin system (RAS) is a master regulatory element for many vital body processes. Besides, its local effect has been described in several body tissues that can affect cellular activity, tissue injury, and sometimes regeneration [22,23,24]. Additionally, the RAS system has been implicated in many different chronic diseases due to physiological alterations that may impact angiotensin peptides, including Ang II, Ang III, Ang IV, Ang 2–10, and Ang (1–7) [25,26,27], and metabolic shifts, whereas inhibiting angiotensin receptors has been proven to enhance glycemic management and lower hepatic triglyceride levels [24,25,26,27,28].

Lantheir et al. [29], demonstrated that selective ablation of Kupffer cells significantly increases high-fat diet-induced hepatic insulin resistance. This confirms its important pathological role in the initiation of insulin resistance. Besides, the inflammatory changes occurring in the adipose tissue, which is responsible for the release of prostaglandin E2 that is involved in the modulation of hepatic glucose output, regulation of cytokine production, and induction of insulin resistance in hepatocytes in collaboration of IL-6, induced a significant increase in Kupffer cells and the pro-inflammatory activation of Kupffer cells [30]. Moreover, an ischemia/reperfusion exposed liver model showed apparent broad hemorrhagic necrosis, extensive areas of portal inflammation, and a moderate increase in inflammatory cell infiltration, and this histological damage was ameliorated by losartan [31]. In addition, a diabetic rat model treated with losartan showed a decrease in the number of inflammatory cells at the liver tissue [32]. These data couple with our histopathological liver data that are shown in Figure 7.

Many studies have shown that ACE 2 plays an important role in insulin resistance [33], and that Ang (1–7) responds to ACE 2 signaling via the MAS receptor [34], with FVB/N Mas-deficient mice exhibiting insulin resistance and glucose intolerance [35], implying that Ang (1–7) signaling is involved in the development of type 2 diabetes and metabolic syndrome [36,37]. In agreement with our present study results, we show a significant decrease in ACE 2/Ang (1–7) in the positive control group (Figure 2), resulting in a hyperinsulinemia as shown in (Figure 1). On the other hand, treated rats exposed to the benzosulfonamide derivative and the pioglitazone reference standard increased ACE 2/Ang (1–7) in hepatocytes cells (Figure 2) that directly proportionally affect insulin secretion, as shown in Figure 1, and glycogen deposition (Figure 2). This coupled with mononuclear inflammatory cell infiltration at the portal area, with hemorrhage in liver section (Figure 7), and significant ill-distinct islets containing vacuolated and necrotic cells (Figure 8) and limited expression of GLUT 2 in the islets of Langerhans (Figure 9). Chai et al. [38] reported that angiotensin receptor 4 (AT4) may modulate glucose uptake by modulating the trafficking of GLUT4. This agree with our findings, as ACE 2/Ang (1–7) levels were corrected through treatment with the synthetic compound modulating pancreatic GLUT 2 expression. Furthermore, Yuan et al. [39] highlight the important significance of Ang (1–7) treatment in improving the pancreatic microcirculation and islet micro-vessel density via endothelial vasodilation.

It has been well established that the insulin receptor activates phosphatidyl 3-kinase and AKT via adaptor proteins, such as insulin receptor substrate 1 (IRS-1) or insulin receptor substrate 2 (IRS-2) [40]. In agreement, Ang (1–7) has been shown to produce similar effects in an insulin resistance rat model subjected to a high fructose concentration in the diet, which significantly induced insulin signaling pathway impairment. In this paradigm, the continuous Ang (1–7) administration improves insulin-stimulated tyrosine phosphorylation of the insulin receptor and IRS-1 as well as Akt Ser473 phosphorylation in skeletal muscles [41,42]. Giani et al. [43] confirmed the significant involvement of Ang (1–7) in stimulating the phosphorylation of JAK 2, IRS 1, and Akt in the rat heart. The AKT pathway is vital in PI3K signal transduction, which is required for insulin transduction and metabolism [40,44]. These findings are in agreement with our study (Figure 2, Figure 3, and Figure 6) which were further supported by the liver and pancreas histopathological study (Figure 7, Figure 8 and Figure 9).

Previous studies inveterate insulin as the primary ligand for the PI3K/AKT pathway, which regulates lipid and glucose metabolism in different essential body organs, such as the brain, liver, muscle, adipose, and pancreas [45]. In the current study data, the PI3K/AKT signaling pathway is upregulated via treatment (Figure 6), increases glucose utilization and decreases gluconeogenesis in the liver tissues (Figure 2), increases body lipid deposition, and, thus, reduces FFA circulation in adipose tissue by regulating leptin and GLP-1, which regulates the balance of lipid and glucose metabolism (Figure 5). Zander et al. [46] state the importance of GLP-1 in enhancing pioglitazone activity, stimulating insulin sensitivity and proinsulin synthesis, and inhibiting glucagon secretion. Furthermore, in an animal model, treatment with GLP-1 ameliorated weight reduction via appetite and glycemic control [47]. In addition, standard pioglitazone is a potent anti-inflammatory, reducing proinflammatory cytokines affecting tumor necrosis factor alpha and pro-coagulant factors [48]. Thus, the thiazolideine pioglitazone group provides cardiovascular protection by reducing tri-glycerides, cholesterol, and raised HDL, resulting in metabolic syndrome control and improved patient blood glucose levels [49]. These previous data support our finding for pioglitazone and the benzosulfonamide pioglitazone derivative in controlling plasma insulin levels (Figure 1), stimulating insulin receptor sensitivity (Figure 3), and reducing inflammatory mediators via upregulation anti-inflammatory ACE 2/Ang (1–7) (Figure 2) as well as reducing IL-1β (Figure 4). On the other hand, controlling appetite via controlling leptin, GLP-1, and glycogen levels (Figure 2 and Figure 5) all coupled with a histopathological confirmation (Figure 7, Figure 8 and Figure 9).

Nov et al. [50] revealed the role of IL-1β in regulating lipid storage capacity in the adipose tissue and liver-mediated autocrine/paracrine action promoting local inflammation and generating fatty liver, which contributes to stenosis and liver insulin resistance. Other findings included a chronic low-grade inflammation in diabetes patients with decreased glucose tolerance, which was caused in part by IL-1β generated by glucose and further hindered insulin secretion [51]. Interestingly, a study found that individuals with impaired fasting glucose levels had a greater between-group rise in Ang (1–7) compared to those with normal glucose levels, while IL-1β increase may reduce Ang (1–7) in the same way that glucose causes impaired insulin secretion [52]. Furthermore, antagonism of the conventional ACE 2/Ang II/AT-1 axis has been observed to shift towards the promotion of the antioxidant and anti-inflammatory pathway ACE2/Ang (1–7)/Mas axis [53]. Side by side, it was reported in an Alzheimer disease rat model that the potential therapeutic strategy of diminazene ameliorates induced hippocampal ACE2/MasR activation with PI3K/Akt transduction endorsed many neuroprotective, anti-apoptotic, and anti-inflammatory effects [54]. Additionally, it was revealed that inducing insulin resistance with STZ and HFD dramatically reduced AMPK levels in HepG2 cells [55]. All these previous studies agreed with our findings that subjecting rats to HFD and STZ injections resulted in hyperinsulinemia (Figure 1), as well as downregulation of the p-PI3K/p-AKT/p-mTOR signaling pathway in the liver (Figure 6), which in turn decreased the sensitivity of IRS-1/IRS-2 (Figure 3), while treatment with pioglitazone and the benzosulfonamide derivative resulted in increasing insulin receptor sensitivity of IRS-1/IRS-2, resulting in an upregulated p-PI3K/p-AKT/p-mTOR signaling pathway, in agreement with the histological results (Figure 7, Figure 8 and Figure 9).

## 4. Materials and Methods

### 4.1. Test Agents, Chemicals, Reagent Kits, and Antibodies

The experiments were conducted using the following materials: streptozotocin (catalog number MFCD00006607), enzyme-linked immunosorbent assay (ELISA) kits for rat angiotensin-converting enzyme II (ACE 2) (catalog number MBS705139), angiotensin (1–7) (catalog number LS-F32295), glucagon-like peptide 1 (GLP-1) (catalog number E-EL-R0059), glycogen (catalog number MBS731185), leptin (catalog number LS-F318), insulin (catalog number MBS 724709), insulin receptor substrate 1 (IRS 1) (catalog number LS-F11840), insulin receptor substrate 2 (IRS 2) (catalog number MBS720661), interleukin 1 beta (catalog number SEA563Ra), the primary antibodies for Western blot assay against t-pi3k (catalog number sc-1637), P-pi3k (catalog number sc-293115), t-AKT (catalog number #3062), P-AKT (catalog number Ser241), t-m-TOR (catalog number #2972), and P-m-TOR (catalog number Ser2448). The above materials were obtained from Santa Cruz Biotechnology (Dallas, TX, USA), All other chemicals, solvents, buffers, and reagents were obtained from authorized sources and were all of analytical grades.

### 4.2. Animals

Adult female albino rats ranging from 200–220 g were kept for adaptation to a 12/12 h cycle before starting the experimental design. Furthermore, after adaptation, the rats were randomized into different groups according to their weight. Then, three groups were fed a high-fat diet. On the other hand, the fourth group were kept as a normal control group that was fed a normal chow composition as described in the experimental design. Animal handling and care were conducted in accordance with the guidelines designated by the references of the National Institutes of Health (NIH) Guide for Care and Use of Laboratory Animals (Publication No. 85-23, revised 1985).

### 4.3. High-Fat Diet (HFD)

Ahmed et al. (2019) [5] have outlined how to make the HFD. In a nutshell, a 1:3 ratio of raw sheep fat lard to feed was used. Wheat flour (7%), glucose (10%), salt combination (6%), bran (4%), vitamin mixture (5000 IU/g, D3: 100 IU/g, B1: 1 mg/g, B2: 1.25 mg/g, B6: 0.5 mg/g, B12: 5 mg/g, C: 15 mg/g, E: 4 mg/g, and K3: 0.75 mg/g) and amino acids (methionine: 25 mg/g and lysine) were used to create a consistent edible paste by adding enough water.

### 4.4. Experimental Design

Rats were distributed randomly into four groups, each with 10 rats. The first group was the normal control group that received vehicles, the second group was the type 2 diabetic control group that received only STZ after the rats were fed a HFD for three weeks, the third group was the reference hypoglycemic group that received pioglitazone (20 mg/kg/day) [56], and the last group was the tested synthetic compound group that received (20 mg/kg/day), a dose similar to the pioglitazone reference hypoglycemic treatment drug. All drugs and vehicles were applied orally for fourteen consecutive days, starting from day twenty-four after the rats were fed a HFD and STZ induction. The animals fasted overnight, and blood and tissue samples were collected in the last day of the experimental design.

### 4.5. General Procedure for the Synthesis of the Benzenesulfonamide Derivative

This is fully described in the Supplement Data (S1).

### 4.6. Method for Type II Diabetes Mellitus Induction

Rats were subjected for metabolic syndrome for three consecutive weeks with a high-fat diet before streptozotocin induction in order to induce insulin resistance. After three weeks, the rats subjected to a single intra-peritoneal injection of 45 mg/kg of freshly prepared streptozotocin dissolved in 0.1 M citrate buffer. Besides, to avoid animal death, 20 percent glucose was added to the drinking water for 48 h after induction with the intra-peritoneal streptozotocin injection [57,58]. At 72 h after streptozotocin induction, diabetic rats were defined as those with blood glucose levels greater than 300 mg/dL as determined by a blood sample from the rat’s tail using an (ACCU-CHEK^®^, Roche, Hague Road, Indianapolis, IN, USA) instrument [59]. The test agent was administrated starting at 24 h after diabetes induction and for 15 consecutive days after metabolic syndrome and STZ-induced T2DM.

### 4.7. Sampling

#### 4.7.1. Blood Sampling

Rats fasted for 8 h overnight before blood samples were obtained through the retro-orbital plexus utilizing heparinized tubes under low-dose anesthesia. The blood was allowed to clot on broken ice for 10 min before centrifugation at 1000× *g* using a cooling centrifuge for 20 min (Sigma 3–30 k, An der Unteren Söse, Osterode am Harz, Germany). The clear serum supernatant layer was wisely withdrawn and stored in a deep freezer at −80 °C (Als Angelantoni Life Science, Località Cimacolle, Massa Martana, Italy) until the time of assay.

#### 4.7.2. Tissue Sampling

Animal were dislocated at the cervical spine, and the liver tissue and pancreas were exposed cautiously and dissected gently free of neighboring tissue. The liver was distributed into two portions and both were well-preserved in freezing media at (−80 °C) until the time of assay for ACE 2, Ang (1–7), IRS 1, IRS 2, Il-1β, leptin, GLP-1, glycogen, p-PI3K, p-AKT, and p-mTOR. The pancreas was fixed in a 10%formalin solution for two days, then the tissue was washed with saline and merged in 10% ethanol until the time of the GLUT 2 immunohistochemical assay and histopathological liver examination.

### 4.8. Assessment of Biomarkers

#### 4.8.1. ELISA of Serum and Tissue Biomarkers

Fasting insulin levels were estimated in the serum, while liver tissue levels of ACE2, Ang (1–7), IRS 1, IRS 2, Il-1β, leptin, GLP-1, and glycogen were measured using ELISA test reagent kits and an ELISA processing system (Model Spectra Max Plus-384 Absorbance Microplate Reader, Philadelphia, Bridgeport, CT, USA) according to the sandwich technique described previously [60,61].

#### 4.8.2. Western Blot Analysis of the PI3k/AKT/mTOR Signaling Pathway

Tissue levels of p-PI3K, p-AKT, and p-mTOR were determined using the Western blot technique described previously [62] using assessments by the BioRad micro protein electrophoresis separation unit (Model 1658004, Sinorica International Patent and Trademark, Germantown, MD, USA). The complete methodology for the assessment was described in the Supplement Data (S2).

#### 4.8.3. Histopathological Study

The preparation of liver tissue slides for staining with standard Hematoxylin and Eosin (H&E) staining for the histopathological research was carried out according to the method published by Banchroft and Steven [63].

##### Immunohistochemical Assay

The pancreas tissue GLUT 2 assay was performed using the immunohistochemistry technique described earlier [64] by incubating primary antibodies against GLUT 2, followed by secondary antibodies, and finally diaminobenzidine/H_2_O_2_ as a chromogen. Hematoxylin counterstaining was used, and the slides were inspected under a light microscope with the assistance of a pathologist.

### 4.9. Statistical Analysis

Means and standard errors of means were used to represent the results (S.E.M). The statistical package for social sciences (SPSS; version 19.0) computer software (SPSS Inc., Chicago, IL, USA) was used to perform one-way analysis of variance (ANOVA) tests followed by Tukey–Kramer tests on biochemical measurements, with a p value of 0.05 considered statistically significant. The Western blot bands were quantified using the Image J computer software (National Institutes of Health (NIH) Rockville Pike, Bethesda, MD, USA).

## 5. Conclusions

In conclusion, the present study demonstrates the hypoglycemic activity of a new synthetic compound benzenesulfonamide derivative that can protect against HFD and STZ-induced type 2 diabetes mellitus in experimental rats, probably via addressing metabolic syndrome, glycogen depositions, insulin sensitivity, and the blood glucose level abnormalities caused by immunological and metabolic assaults. These effects are mediated by upregulation of ACE 2/Ang (1–7)/PI3K/AKT/mTOR, glycogen, IRS 1, IRS 2, and GLUT 2, coupled with significantly improved serum insulin levels and the suppression of IL-1β. All of these findings indicate that benzenesulfonamide derivatives have a hypoglycemic modifying effect, which bodes well for future clinical trials.

## Data Availability

Data is contained within the article or Supplementary Materials.

## Supplementary Materials

The following supporting information can be downloaded at: https://www.mdpi.com/article/10.3390/ph15030341/s1, S1: General procedure for the synthesis of benzene sulfonamide derivative; S2: Western blot analysis technique of PI3k/AKT/mTOR signaling pathway.

## Abbreviations

Abbreviation	Meaning
ACE	Angiotensin-Converting Enzyme
ACE 2	Angiotensin-Converting Enzyme 2
Akt	Serine/Threonine Kinase
Ang (1–7)	Angiotensin (1–7)
Ang II	Angiotensin 2
ANOVA	Analysis Of Variance
AT 1	Angiotensin II Receptor Type 1
DM	Diabetes Mellitus
ELISA	Enzyme-Linked Immunoassay
FFA	Free Fatty Acid
GLP-1	Glucagon-Like Peptide 1
GLUT 4	Insulin-Regulated Glucose Transporter-4
H&E	Hematoxylin And Eosin
HDL	High-Density Lipoprotein
HFD	High-Fat Diet
IL 6	Interleukin 6
IL-1β	Interleukin 1 Beta
IL-1β	Interleukin 1 Beta
IRS 1	Insulin Receptor Substrate 1
IRS 2	Insulin Receptor Substrate 2
JAK 2	Janus Kinase 2
mTOR	Mammalian Target of Rapamycin
NAFLD	Non-Alcoholic Fatty Liver Disease
PI3K	Phosphoinositide 3-Kinases
RAS	Renin-Angiotensin System
SPSS	Statistical Analysis Software
STZ	Streptozotocin
