# Supplementary material for: Pioglitazone Synthetic Analogue Ameliorates Streptozotocin-Induced Diabetes Mellitus through Modulation of ACE 2/Angiotensin 1–7 via PI3K/AKT/mTOR Signaling Pathway
Source: Pharmaceuticals (Basel). 2022 Mar 10;15(3):341. doi: 10.3390/ph15030341 (PMC8955304; doi:10.3390/ph15030341)
Supplement: Supplementary file 1 [file pharmaceuticals-15-00341-s001.zip › pharmaceuticals-1583529-supplementary.pdf]

Pioglitazone Synthetic Analogue Ameliorates Streptozotocin-Induced Diabetes Mellitus through Modulation of ACE 2/Angiotensin 1-7 via PI3K/AKT/mTOR Signaling Pathway

Yasmin Moustafa Ahmed [1], Mohamed A. Abdelgawad [2*], Khaled Shalaby [3], Mohammed M. Ghoneim [4], Asmaa M. AboulMagd [5*], Nada S. Abdelwahab [5,6], Hossam M. Hassan [7] and Asmaa M. Othman [8]

[1] Department of Pharmacology and Toxicology, Faculty of Pharmacy, Nahda University, Beni-Suef, Egypt.
[2] Department of Pharmaceutical Chemistry, Collage of Pharmacy, Jouf University, Sakaka, Al Jouf 72341,Saudi Arabia.
[3] Department of Pharmaceutics, Collage of Pharmacy, Jouf University, Sakaka, Al Jouf 72341, Saudi Arabia.
[4] Department of Pharmacy Practice, College of Pharmacy, AlMaarefa University, Ad Diriyah 13713, Saudi Arabia.
[5] Department of Pharmaceutical Chemistry, Faculty of Pharmacy, Nahda University (NUB), Beni-Suef, Egypt.
[6] Department of Pharmaceutical Analytical Chemistry, Faculty of Pharmacy, Beni-Suef University,
Beni-Suef, Egypt.
[7] Department of Pharmacognosy, Faculty of Pharmacy, Beni-Suef University, Beni-Suef, Egypt.
[8] Department of Internal Medicine, Faculty of Medicine, Beni-Suef University, Beni-Suef 62514, Egypt.
* Correspondence: Prof. Dr. Mohamed A. Abdelgawad (mhmdgwd@ju.edu.sa), Dr. Asmaa M. AboulMagd (asmaa.aboulmaged@nub.edu.eg)

General procedure for the synthesis of benzenesulfonamide derivative.

To a solution of 4-oxo-4,5-dihydrothiazol-2-yl amino benzenesulfonamide (0.271gm, 0.001 mol) in glacial acetic acid (20 mL), sodium acetate (0.164gm, 0.002 mol) and vanillin (0.02 mol) was added. The whole mixture was refluxed for 48 h and the reaction was monitored by TLC till completion of the reaction. The solid resulted was filtered and washed from ethanol several times. The precipitate was crystallized from acetic acid to afford the title compound.

Orange solid: yield, 82%; m.p. 263±2 °C; IR (KBr) ν_{max}:) 3561 (OH), 3360, 3271 (NH_2), 3201 (NH), 2862 (CH aliph.), 1678 (C=O) cm^{-1}, ^{1}H-NMR (DMSO-d_6) δ: 3.97 (s, 3H, OCH_3), 6.95 (d, *J*= 8.0 Hz, 1H, Ar-H), 7.35 (s, 1H, CH), 7.38 (m, 3H, Ar-H), 7.50 (s, 1H, OH), 7.55 (d, *J*= 8.0 Hz, 1H, Ar-H), 7.65 (d, *J*= 8.0 Hz, 1H, Ar-H), 7.84 (d, *J*= 8.0 Hz, 1H, Ar-H), 7.92 (s, 1H, NH), 7.99 (s, 2H, NH_2) ppm. ^{13}C-NMR (DMSO-d_6) δ: 57.0, 116.8, 117.0, 117.2, 122.0, 124.4, 127.0, 127.5, 129.1, 132.5, 133.2, 134.5, 136.4, 140.3, 144.9, 160.0, 160.8 ppm. **MS** (Mwt.: 405.05): m/z 405.44 (M+, 58.86%), 476.91 (47.73%), 352.81 (96.34%), 329.33 (61.38%), 284.90 (100%); Anal. Calcd. For $C_{17}H_{15}N_3O_5S_2$: C, 50.36; H, 3.73; N, 10.36. Found: C, 50.22; H, 3.94; N, 10.54 %.

Western blot analysis technique of PI3k/AKT/mTOR signaling pathway

Briefly, a portion of the liver tissue was exposed to RIPA lysis buffer, and then the lysed samples were brought to complete protein extraction. The lysate was kept in ice for 30 min on shaker and cell debris was removed by centrifugation at 16,000 *xg* using a cooling centrifuge. Supernatants were transferred to new tubes for protein concentration determination. Protein separation by gel electrophoresis was performed using the procedure termed SDS-PAGE (sodium dodecylsulfate-polyacrylamide gel electrophoresis) by the support of the appropriate antibodies, where incubation was done overnight in each primary antibody solution against the blotted target protein at 4°C. The blot was rinsed 3-5 times for 5 min with TBST. Incubation was done in the HRP-conjugated secondary antibody solution against the blotted target protein for 1 hr at room temperature. The blot was rinsed 3-5 times for 5 min with TBST. The chemiluminescent substrate was applied to the blot according to the manufacturer's recommendation. The chemiluminescent signals were captured using a CCD camera-based imager. Image analysis software was used to read the band intensity of the target proteins against control sample after normalization by *beta* actin on the Chemi- Doc MP imager.
